# Inhibition of Fatty Acid Synthase Reduces Blastocyst Hatching through Regulation of the AKT Pathway in Pigs

**DOI:** 10.1371/journal.pone.0170624

**Published:** 2017-01-20

**Authors:** Jing Guo, Nam-Hyung Kim, Xiang-Shun Cui

**Affiliations:** Department of Animal Sciences, Chungbuk National University, Chungbuk, Cheongju, Republic of Korea; Institute of Zoology Chinese Academy of Sciences, CHINA

## Abstract

Fatty acid synthase (FASN) is an enzyme responsible for the *de novo* synthesis of long-chain fatty acids. During oncogenesis, FASN plays a role in growth and survival rather than acting within the energy storage pathways. Here, the function of FASN during early embryonic development was studied using its specific inhibitor, C75. We found that the presence of the inhibitor reduced blastocyst hatching. FASN inhibition decreased *Cpt1* expression, leading to a reduction in mitochondria numbers and ATP content. This inhibition of FASN resulted in the down-regulation of the AKT pathway, thereby triggering apoptosis through the activation of the p53 pathway. Activation of the apoptotic pathway also leads to increased accumulation of reactive oxygen species and autophagy. In addition, the FASN inhibitor impaired cell proliferation, a parameter of blastocyst quality for outgrowth. The level of OCT4, an important factor in embryonic development, decreased after treatment with the FASN inhibitor. These results show that FASN exerts an effect on early embryonic development by regulating both fatty acid oxidation and the AKT pathway in pigs.

## Introduction

Fatty acid synthase (FASN) is a key enzyme catalyzing the *de novo* synthesis of long-chain fatty acids from acetyl-CoA and malonyl-CoA. Fatty acids (FAs) are essential constituents of lipids involved in membrane biogenesis and are critical substrates in energy metabolism. There are two sources of FAs: exogenous FAs and endogenous FAs. The biosynthesis of endogenous FAs is catalyzed by FASN[1, 2]. The synthesis of FAs by FASN is initiated by the conversion of acetyl-CoA to malonyl-CoA. Malonyl-CoA is then used for FA synthesis and is involved in elongation[3]. FAs are important constituents of sphingolipids, ceramides, and glycolipids and are involved in many biological processes[4]. Under normal conditions, FASN-synthesized FAs are stored as triacylglycerols and are catabolized through FA oxidation (FAO) when necessary[5]. *De novo* FA synthesis is very active during embryogenesis and plays a critical role in embryonic development[6].

In some cases, FASN contributes to growth and survival rather than the energy storage pathway. FASN inhibition impairs DNA replication, causing cell cycle arrest before the G1 phase through mechanisms involving p21, p27, BRCA1, and NFκB[7, 8]. Furthermore, FASN inhibition induces tumor cell apoptosis through the down-regulation of AKT and suppression of p53 function[9, 10]. In addition, during the menstrual cycle, FASN and E_2_-ER signaling control endometrial cell proliferation[11].

FASN studies primarily focus on its role in cancer biology. Thus, the function of FASN in early embryonic development is poorly understood. In this study, C75, a pharmacological inhibitor of FASN, was used to study the role of FASN in embryogenesis. C75 is a cerulenin-derived synthetic FASN inhibitor and has been used in many previous studies [12, 13]. C75 inhibits purified mammalian FASN by blocking its KS domain[14]. Specific depletion of FASN by RNAi leads to loss of sensitivity to C75, confirming that C75-induced damage is dependent on inhibition of FASN activity[9, 10]. Here, we hypothesized that FASN might be involved in porcine embryonic development either through its action in lipid metabolism or through other pathways. C75 was used to determine the function of FASN in embryogenesis and to elucidate the mechanisms involved. Our results show that FASN plays critical roles during embryonic development *via* its regulatory functions in FA synthesis and the AKT pathway.

## Materials and Methods

All chemicals used in this study were purchased from Sigma-Aldrich (St. Louis, MO, USA), unless otherwise indicated.

### 2.1. Oocyte collection, in vitro maturation, and embryo culture

All animal studies were performed in strict accordance with institutional guidelines and prior approval was obtained from the Institutional Animal Care and Use Committee (IACUC) of Chungbuk National University.

Ovaries from prepubertal gilts were obtained from a local slaughterhouse and transported in saline at 37°C to the laboratory. Follicles 3–6 mm in diameter were aspirated. Cumulus-oocyte complexes (COCs) surrounded by more than three layers of cumulus cells were selected for culture[15]. COCs were isolated from follicles and washed three times in TL-HEPES. COCs were cultured in tissue culture medium 199 (TCM 199) supplemented with 10% porcine follicular fluid, 0.1 g/L sodium pyruvate, 0.6 mM L-cysteine, 10 ng/mL epidermal growth factor, 10 IU/mL luteinizing hormone, and 10 IU/mL follicle stimulating hormone at 38.5°C for 44 h in a humidified atmosphere of 5% CO_2_. After maturation, cumulus cells were removed by treatment with 0.1% hyaluronidase and repeated pipetting. For activation of parthenogenesis, oocytes with polar bodies were selected, activated by two direct current pulses of 1.1 kV/cm for 60 μs, and then incubated in porcine zygote medium (PZM-5) containing 7.5 μg/mL of cytochalasin B for 3 h. Finally, embryos were cultured in PZM-5 for 8 d at 38.5°C in a humidified atmosphere with 5% CO_2_. On the 5^th^ day, fetal bovine serum was added to the medium for a final concentration of 10%. To determine the effect of FASN on early porcine embryonic development after embryo activation, the FASN inhibitor C75 was added to the medium at final concentrations of 10 or 20 μM. The 10-μM concentration was used in the following experiments as it represents the minimum concentration inducing an effect on blastocyst formation.

### 2.2. ATP content assay

The ATP contents from 30 blastocysts per treatment group were measured using an ATP Determination Kit (Invitrogen, Carlsbad, CA, USA). Briefly, samples were washed three times with PBS and then transferred individually into 1.5-mL tubes. Media were removed and blastocysts were frozen and thawed for lysis. Approximately 100 μL of ice-cold somatic cell reagent (FL-SAR) was added to each tube, and samples were incubated in an ice-water bath for 5 min. Thereafter, 100 μL of ice-cold assay buffer (diluted 1:25 with ATP assay buffer, FL-AAB) was added and the tubes were maintained at room temperature for 5 min under limited light conditions. The ATP concentration was measured using a luminometer (Berthold, Wildbad, Germany) with a sensitivity of 0.01 pmol. The ATP concentration in the control group was arbitrarily set at 1. Three separate experiments were performed.

### 2.3. Membrane potential assay and mitochondrial copy number analysis

Day-8 blastocysts were washed three times with phosphate buffered saline/polyvinyl alcohol (PBS/PVA) and incubated in culture medium containing 1 mM 5,5’,6,6’-tetrachloro-1,1’,3,3’-tetraethyl-imidacarbocyanine iodide (JC-1) (Invitrogen) at 37°C in a humidified atmosphere of 5% CO_2_ for 30 min. Membrane potential was calculated as the ratio of red florescence, which corresponds to activated mitochondria (J-aggregates), to green fluorescence, which corresponds to less-activated mitochondria (J-monomers)[16]. Fluorescence was visualized with an epifluorescence microscope (Nikon Corp., Tokyo, Japan). The fluorescence intensity in the control group was arbitrarily set at 1, and the fluorescence intensities in the treatment groups were measured and expressed as relative values with respect to the control group. Three separate experiments were performed, and 10 blastocysts were examined per experiment.

Total DNA was isolated from 10 blastocysts using the Puregene DNA Isolation Kit (Invitrogen) according to the manufacturer’s instructions. Blastocyst DNA samples were then used for real-time polymerase chain reaction (PCR) experiments. The primers *Ndufaf3* and *Gapdh* are described in a previous study[17]. The reactions were performed as follows: 95°C for 3 min followed by 40 cycles of 95°C for 15 s, 60°C for 20 s, and 72°C for 20 s, and a final extension at 72°C for 5 min. The relative quantification of the mitochondrial copy numbers was performed using the 2^-ΔΔCt^ method. Three separate experiments were performed, with three replicates per experiment.

### 2.4. Reactive Oxygen Species (ROS) staining

Blastocysts were incubated for 15 min in IVC medium containing 10 μM 2’,7’-dichlorodihydrofluorescein diacetate (H_2_DCF-DA) at 37°C. After incubation, 30 blastocysts per group were washed three times in IVC medium and transferred to PBS drops covered with paraffin oil in a polystyrene culture dish. The fluorescent signal was captured using an epifluorescence microscope. The fluorescence intensity in the control group was arbitrarily set at 1, and the fluorescence intensity in the treatment group was measured and expressed as a relative value with respect to the control group.

### 2.5. Terminal deoxynucleotidyltransferase-mediated 2'-deoxyuridine 5'-triphosphate (dUTP) nick-end labeling (TUNEL) assay

The blastocysts were collected after C75 treatment, fixed in 3.7% paraformaldehyde for 15 min at room temperature, and permeabilized in 0.5% Triton X-100 for 1 h at 37°C. The embryos were incubated with fluorescein-conjugated dUTP and the terminal deoxynucleotidyltransferase enzyme (*In Situ* Cell Death Detection Kit, Roche; Mannheim, Germany) for 1 h at 37°C. The embryos were washed three times in PBS/PVA, treated with Hoechst 33342 for 5 min, washed three times in PBS/PVA, and mounted onto glass slides. Images were captured using a confocal microscope (Zeiss LSM 710 META, Jena, Germany).

### 2.6. Immunofluorescence and confocal microscopy

Embryos were fixed in 3.7% paraformaldehyde for 20 min at room temperature, permeabilized with PBS/PVA containing 0.5% Triton X-100 at 37°C for 1 h, and then incubated in PBS/PVA containing 3.0% bovine serum albumin at 37°C for 1 h. Subsequently, the embryos were incubated overnight at 4°C with anti-LC3 (ab58610, 1:100; Abcam, Cambridge, UK), anti-cytochrome C (ab110325, 1:100; Abcam, Cambridge, UK), anti-pAKT (9271, 1:100; Cell Signaling Technology, Danvers, MA, USA), anti-p53 (sc6243, 1:100; Santa Cruz, CA, USA), or anti-OCT4 (sc8628, 1:100; Santa Cruz, CA, USA) antibodies. After washing three times in PBS/PVA, the oocytes and embryos were incubated at 37°C for 1 h with either goat anti-rabbit IgG or rabbit anti-goat IgG. The oocytes and embryos were then stained with Hoechst 33342 for 5 min, washed three times in PBS/PVA, mounted onto slides, and examined using a confocal microscope (Zeiss LSM 710 META, Jena, Germany). Images were processed using Zen software (version 8.0, Zeiss, Jena, Germany).

### 2.7. 5-Bromo-deoxyuridine analysis

The rate of cell proliferation was analyzed using 5-bromo-deoxyuridine (BrdU). Blastocysts were incubated in 100 μM BrdU for 6 h and then washed 3 times in PBS/PVA. Embryos were fixed in 3.7% paraformaldehyde for 20 min at room temperature, permeabilized with PBS/PVA containing 0.5% Triton X-100 at 37°C for 30 min, and treated with 1N HCl at room temperature for 30 min. Blastocysts were incubated in PBS/PVA containing 3.0% bovine serum albumin at 37°C for 1 h and then with anti-BrdU at 4°C overnight. After washing in PBS/PVA 5 times, blastocysts were incubated at 37°C for 1 h with goat anti-mouse antibody. Finally, blastocysts were stained with Hoechst 33342 for 5 min, mounted onto slides, and examined with a confocal microscope.

### 2.8. Real-time reverse transcriptase-polymerase chain reaction (RT-PCR)

Day-8 blastocysts were collected. mRNA was extracted from 10 blastocysts per group using a DynaBeads mRNA Direct Kit (Dynal Asa, Oslo, Norway) according to the manufacturer’s instructions. cDNA was obtained by reverse transcription of mRNA using the Oligo(dT)12-18 primer and SuperScript III Reverse Transcriptase (Invitrogen Co., Grand Island, NY, USA). The amplification cycles were as follows: 95°C for 3 min followed by 40 cycles of 95°C for 15 s, 60°C for 30 s, and 72°C for 20 s, and a final extension at 72°C for 5 min. The primers used in the study were listed in Table 1. Relative gene expression was normalized to internal porcine *Gapdh* mRNA level using the 2^-ΔΔCt^ method.

**Table 1:** List of primers used for real-time RT-PCR.

Gene	Primer Sequence(5’-3’)	Annealing Temperature(°C)	Product size (bp)
***Fn1***	F:AGGGCGATGAACCACAGT	60	221
	R:GCTCCAGCGAACGACAAT		
***Cox2***	F:GGCTGCGGGAACATAATAGA	55	183
	R:GCAGCTCTGGGTCAAACTTC		
***Cpt1***	F: TTATCCACCAGCCAGACC	55	203
	R: CCGAAGCGATGAGAATCC		
***Mnsod***	F:AAGTTGACCGCTGTATCC	60	128
	R:AGACCTGTTGTTCCTTGC		
***Gpx1***	F:CTCGGTGTATGCCTTCTC	60	139
	R:ATTCATCTGGGTGAGTCC		
***Tfam***	F:GGTTTTCCAAAGAAGCCTATGAC	60	182
	R:TGCCAGTCTGCCCTATAAGC		
***Bcl2***	F:GCCGAAATGTTTGCTGAC	60	154
	R:GCCGATCTCGAAGGAAGT		
***Bcl-xl***	F: AGGGACAGCGTATCAGAG	60	277
	R: TGGTCATTCAGGTAAGTGG		
***Casp3***	F:GACGGACAGTGGGACTGAAGA	60	101
	R:GCCAGGAATAGTAACCAGGTGC		
***Oct4***	F: CCCCGCCCTATGACTTCT	60	269
	R: TAGGAGCTTGGCAAATTGTTC		
***Nanog***	F: CTCTCCTCTTCCTTCCTC	55	139
	R: CTTCTGCTTCTTGACTGG		
***Sox2***	F: CGCAGACCTACATGAACG	55	103
	R: TCGGACTTGACCACTGAG		
***Gapdh***	F: TTCCACGGCACAGTCAAG	60	117
	R: ATACTCAGCACCAGCATCG		

### 2.9. Quantitative RT-PCR for microRNA analysis

microRNA (miRNA) was obtained from day-8 blastocysts using the High Capacity cDNA Reverse Transcription Kit (Applied Biosystems, Waltham, MA, USA). Ten blastocysts per group were lysed in RT master mix containing 3.61 μL of H_2_O, 0.5 μL of 10× RT Buffer, 5-plex stem-loop RPs, and 0.065 μL of RNase inhibitor, followed by incubation at 95°C for 5 min. The RT mixture contained 4.3 μL of RNA, 0.065 μL of RNase inhibitor, 0.335 μL of MMLV RT, and 0.25 μL of dNTP RT-PCR. The amplification was performed using the following steps: 16°C for 30 min, followed by 60 cycles of 20°C for 30 s, 42°C for 30 s, and 50°C for 1 s, and a final step at 85°C for 5 min. Quantitative RT-PCR for each miRNA was carried out in 20-μL reaction mixtures that included 1 μL of RT product, 2× SYBR mix, 1 μL of primers, and 8 μL of H_2_O. Amplification parameters used for quantitative RT-PCR were consistent with the manufacturer’s protocol (KAPA Biosystems, Korea). The primers used in this study were listed in Table 2. miRNA expression was normalized to internal U6 level using the 2^-ΔΔCt^ method.

**Table 2:** List of primers used for real-time RT-PCR.

Primer Name	Primer Sequence(5’-3’)
miR-19	F:ACACTCCAGCTGGGTGTGCAAATCCATGCAA
	RT:CTCAACTGGTGTCGTGGAGTCGGCAATTCAGTTGAGACAGTTTT
miR-92	F:ACACTCCAGCTGGGTATTGCACTTGTCCCG
	RT:CTCAACTGGTGTCGTGGAGTCGGCAATTCAGTTGAGACAGGCCG
Let7a	F:ACACTCCAGCTGGGTGAGGTAGTAGGTTGT
	RT:CTCAACTGGTGTCGTGGAGTCGGCAATTCAGTTGAGAACTATAC
U6	F:GCTTCGGCAGCACATATACTAAAAT
	R:CGCTTCACGAATTTGCGTGTCAT
Reverse Primer	CTCAAGTGTCGTGGAGTCGGCAA
	RT: CGCTTCACGAATTTGCGTGTCAT

### 2.10. Statistical analysis

All data were analyzed using the one-way analysis of variance and Chi-square test embedded in Statistical Package for the Social Sciences (SPSS). Each experiment was performed in triplicate and differences were considered significant if P < 0.05.

## Results

### 3.1 Depletion of FASN impairs porcine embryonic development

**Fig 1:**
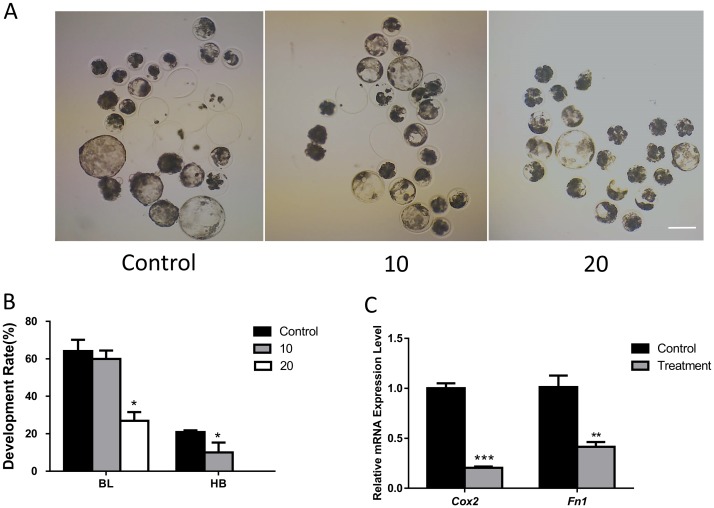
Effect of FASN inhibition on porcine early embryonic development. (A) Effect of increasing FASN inhibitor concentrations on embryonic development. (B) Blastocyst formation and hatching rates. (C) mRNA expression of hatching-related genes is shown in the presence or absence of the FASN inhibitor. *P < 0.05, **P < 0.01, ***P < 0.001. BL:Blastocyst; HB: Hatching Blastocyst. Scale bar: 200μm.

To understand the role of FASN during porcine early embryonic development, we used its inhibitor, C75. Parthenotes were treated with C75 at different doses. As shown in Fig 1A and 1B, there was no significant difference in blastocyst development rates between the control and the 10-μM treatment group (59.55 ± 7.47% vs. 47.52 ± 12.40%). However, blastocyst development decreased significantly in the 20-μM C75 treatment group (25.90 ± 6.23%). In addition, the rate of blastocyst hatching was significantly lower in the presence of 10 μM and 20 μM C75 than in the control group (, 12.48 ± 0.46%,0 vs 22.90 ± 0.08%). Therefore, FASN was confirmed as a critical component in early embryonic development. The expressions of hatching-related genes were also reduced in the treatment groups compared with those in the control (Fig 1C). A FASN inhibitor concentration of 10 μM was used for further experiments.

### 3.2 Inhibition of FASN affects FAO

**Fig 2:**
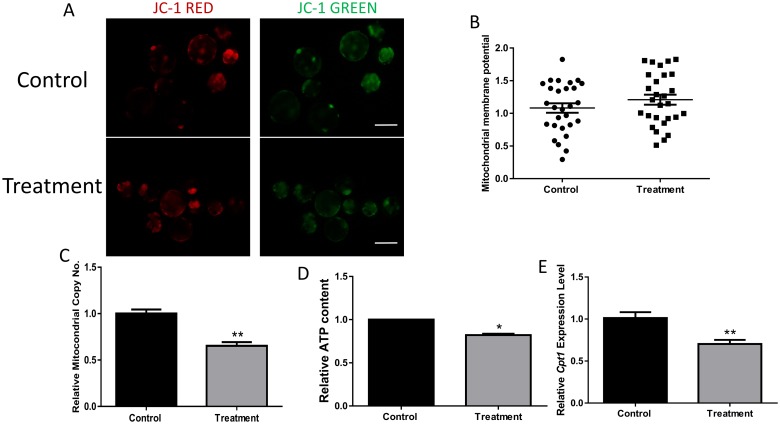
Effect of the FASN inhibitor on fatty acid oxidation. (A) Red fluorescence corresponds to activated mitochondria and green fluorescence corresponds to less-activated mitochondria. (B) Membrane potential was measured as the ratio of red fluorescence to total fluorescence. (C) The relative mtDNA copy number is shown. (D) The ATP content was measured in both the control and treatment groups. (E) The mRNA expression level of Cpt1 is shown in the presence or absence of C75. *P < 0.05, **P < 0.01. Scale bar: 200μm.

Because of the important role of FASN in FA synthesis, the ATP content was assessed after the inhibition of FASN. First, the mitochondrial membrane potential and copy number were examined. No significant differences in membrane potential were observed following the addition of the FASN inhibitor (Fig 2A and 2B). However, the mitochondrial copy number was significantly lower after treatment with the FASN inhibitor (Fig 2C). We also detected a lower ATP content in the control group than in the FASN-inhibitor group (P < 0.05), as shown in Fig 2D. *Cpt1* is the limiting factor in FAO and is a target of FASN. The mRNA expression level of Cpt1 also decreased when embryos were incubated with the FASN inhibitor, which further confirmed the role of FASN in FAO (Fig 2E). Therefore, FASN can affect FAO through the regulation of Cpt1.

### 3.3 Inhibition of FASN increases ROS

**Fig 3:**
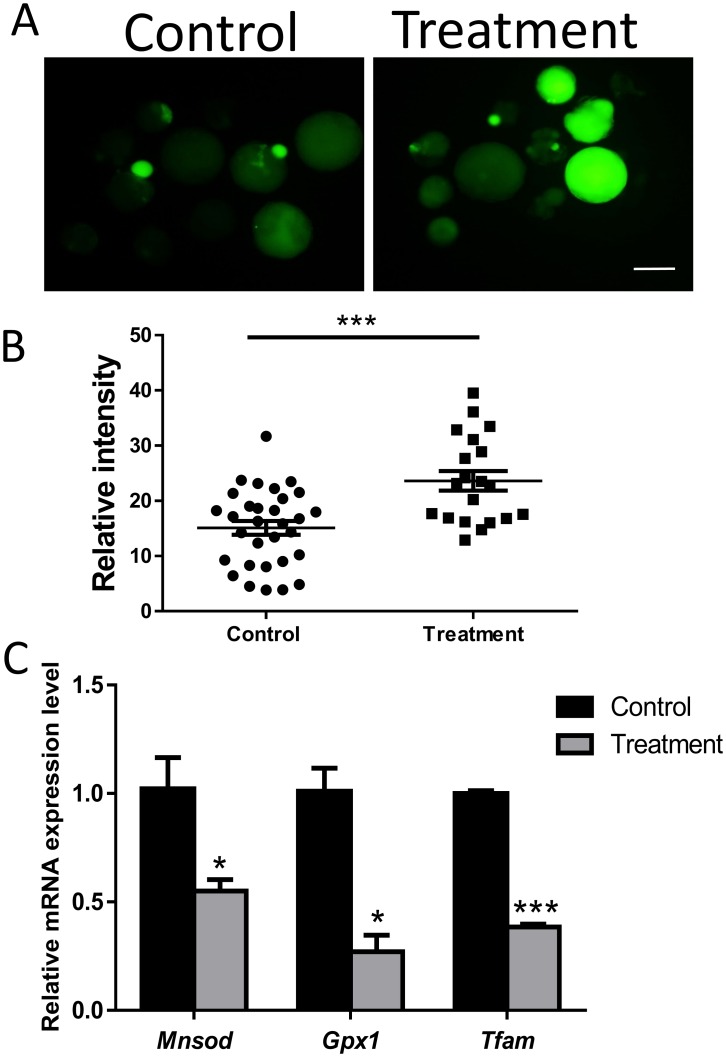
FASN inhibition causes an increase in ROS. (A) ROS staining in porcine blastocysts for various concentrations of FASN inhibitor. (B) Green fluorescence intensity was measured. (C) mRNA expression of Gpx1, Mnsod, and Tfam in blastocysts with or without FASN inhibitor. *P < 0.05, ***P < 0.001. Scale bar: 200μm.

The inhibition of FASN has a negative effect on FAO, and FAO can induce ROS formation. Embryos were treated with H2DCF-DA to determine the impact of FASN inhibition on ROS production. As shown in Fig 3A and 3B, the fluorescence intensity significantly increased after treatment with C75 (P < 0.001), indicating an increase in oxidation activity. The mRNA expression levels of ROS-related genes were examined. *Mnsod*, *Gpx1*, and *Tfam* showed significantly lower expression in treated blastocysts than in control blastocysts (Fig 3C), which confirmed the impact of FASN inhibition on ROS production.

### 3.4 Inhibition of FASN induces cell apoptosis

**Fig 4:**
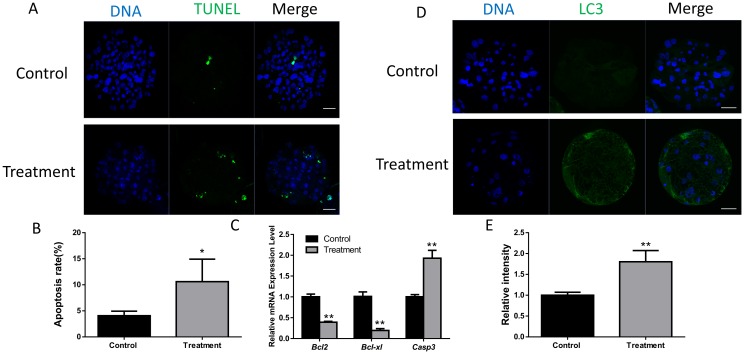
Effect of FASN inhibition on apoptosis and autophagy. (A) TUNEL-positive cells were detected in the presence or absence of the FASN inhibitor. (B) The apoptotic rate is shown. (C) mRNA expression of Bcl2, Bcl-xl, and Casp3 in blastocysts with or without FASN inhibitor. (D) Laser scanning confocal microscopy images of immunostaining for the LC3 protein in porcine blastocysts after treatment with the FASN inhibitor. (E) Relative fluorescence intensity of LC3 is shown. *P < 0.05, **P < 0.01. Scale bar: 50μm.

The embryonic development is influenced by apoptosis; therefore, we checked whether FASN could affect the apoptosis process. The rate of apoptosis was calculated as the ratio between the number of TUNEL-positive nuclei and the total cell number of nuclei. The rate of apoptosis increased significantly in the FASN-inhibitor treated group (Fig 4A and 4B). We also observed decreased expression of the anti-apoptotic genes *Bcl2* and *Bcl-xl*, and the increase of the apoptotic gene *Casp3* (Fig 4C), suggesting that FASN may have a role in modulating apoptosis during embryogenesis. Furthermore, apoptosis can induce cell autophagy. The expression level of LC3 was examined after treatment with the FASN inhibitor. Immunofluorescence staining showed a significant decrease in fluorescence intensity and the mRNA expression level of LC3 was also significantly reduced (Fig 4D and 4E).

### 3.5 FASN inhibition causes release of mitochondrial cytochrome C

**Fig 5:**
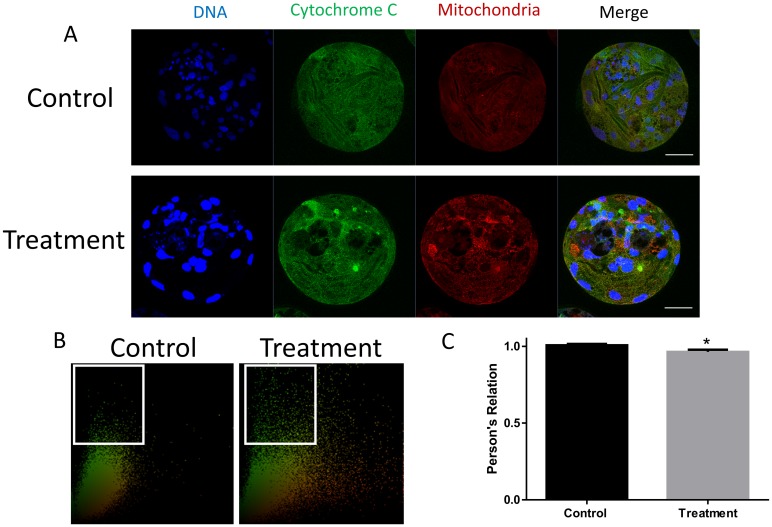
Effect of FASN inhibition on the co-localization of cytochrome C and mitochondria. (A) The localizations of both cytochrome C and mitochondria detected by immunostaining. (B) The co-localization of cytochrome C and mitochondria is shown. (C) The level of co-localization was examined using Pearson’s correlation. *P < 0.05. Scale bar: 50μm.

Cytochrome C is a component of the electron transport chain in mitochondria. The release of cytochrome C from the mitochondria into the cytoplasm is involved in the initiation of apoptosis. The increased release of cytochrome C into the cytoplasm following C75 treatment was assessed by colocalization of cytochrome C with the mitochondria and is shown in Fig 5. Taken together, these data show that the inhibition of FASN causes the release of cytochrome C, which induces the initiation of apoptosis.

### 3.6 FASN influences apoptosis through the AKT pathway

**Fig 6:**
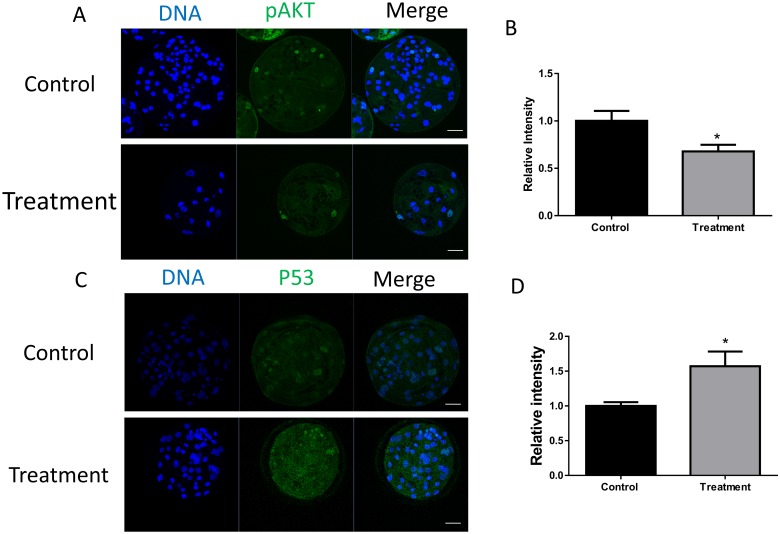
FASN inhibition triggers the activation of the AKT-p53 pathway. (A) Staining of pAKT showed activation and expression of pAKT. (B) The relative intensity of pAKT is shown as bars. (C) Laser scanning confocal microscopy images of immunostaining of p53. (D) Relative intensity of p53 was measured. *P < 0.05. Scale bar: 50μm.

AKT is an important target of FASN, and FASN regulates a number of biological processes through the AKT pathway. We hypothesized that the inhibition of FASN induces apoptosis through the AKT pathway. Immunofluorescence staining showed that pAKT protein levels decreased after the parthenotes had been treated with the FASN inhibitor (Fig 6A and 6B). p53 is a downstream product of the AKT pathway and an increased expression of p53 results in poor developmental potential. We observed that p53 protein levels increased significantly in the FASN inhibitor-treated group (Fig 6C and 6D). Therefore, these results suggest that the inhibition of FASN induces apoptosis through the AKT pathway.

### 3.7 FASN inhibition decreases cell proliferation

**Fig 7:**
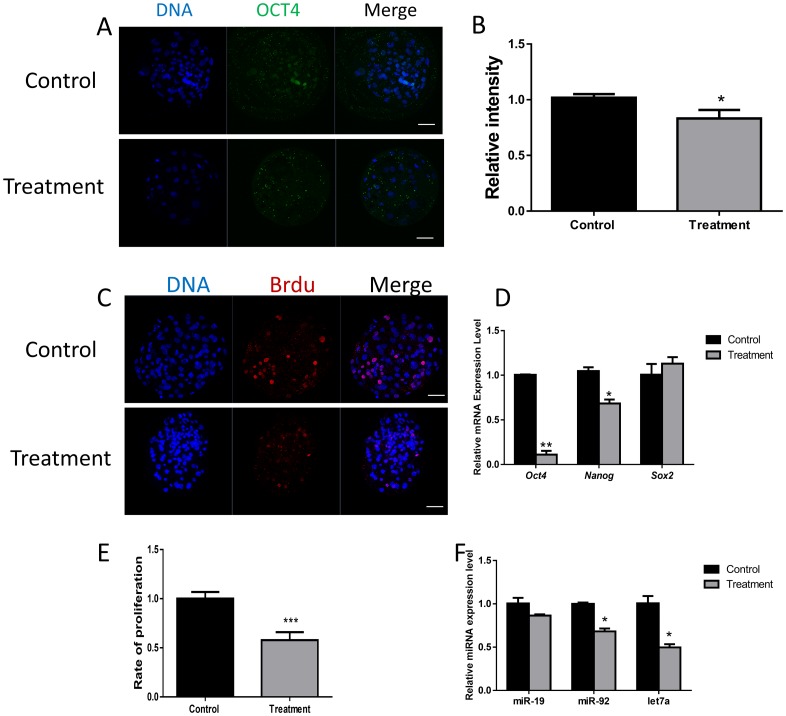
Effect of FASN inhibition on pluripotency. (A) Laser scanning confocal microscopy images of OCT4 protein immunostaining in porcine blastocysts with or without FASN inhibitor. (B) Relative intensity of p53 was measured. (C) Relative Oct4, Nanog, and Sox2 mRNA expression levels are shown as bars. (D) BrdU incorporation in blastocysts after treatment with the FASN inhibitor was examined. (E) Graph summarizing relative proliferation rates. (F) Expression of microRNAs after treatment with the FASN inhibitor. Data are presented as relative expression levels. *P < 0.05, **P < 0.01, ***P < 0.001. Scale bar: 50μm.

AKT-mediated phosphorylation may affect the expression of *Oct4*, an important gene in embryonic development[18]. FASN inhibition led to a decrease in OCT4 expression (Fig 7A, 7B and 7C). The *Oct4* gene is a marker of pluripotency. The expression levels of other pluripotency-related genes were also evaluated. The inhibition of FASN caused a reduction in the expression of *Nanog*, but had no effect on *Sox2* (Fig 7C). These results indicate that FASN inhibition may influence OCT4 expression *via* the AKT pathway, and may also affect pluripotency.

The AKT pathway also influences cell proliferation. Therefore, the effect of FASN on proliferation was measured through BrdU staining (Fig 7D). The percentage of cells undergoing DNA synthesis was calculated by dividing the number of BrdU positive nuclei by the total cell number. As shown in Fig 7E, the percentage of proliferative cells was lower in the presence of FASN inhibitor. Therefore, FASN inhibition can block cell proliferation through the AKT pathway.

To investigate the relationship between FASN and miRNA content, the expression of implantation-related miRNAs was determined. The miR-19 level remained unchanged after treatment with the FASN inhibitor, whereas the levels of both miR-92 and miR-let7a decreased significantly (Fig 7F).

## Discussion

Our results showed that FASN inhibition blocks porcine embryonic development by causing a decrease in ATP content and inducing apoptosis. Therefore, we have shown that FASN acts as a critical regulator of blastocyst formation and hatching. We describe a molecular mechanism by which the FASN inhibitor induces the decrease in FA through the down-regulation of *Cpt1*. In addition, the regulation of apoptosis by FASN occurs *via* the AKT-p53 pathway.

FAs provide twice as much ATP as carbohydrates. FASN-synthesized FAs can be utilized through FAO[5]. In this study, FASN inhibition caused a decrease in ATP content through *Cpt1* down-regulation during embryonic development. ATP content is paramount for embryonic development through its regulation of mitosis, blastocyst formation, and hatching [19–21]. The inhibition of FASN leads to the accumulation of malonyl-CoA, which suppresses Cpt1 during FAO[22]. Cpt1 is the rate-limiting enzyme in FAO, and enables the transport of long-chain FA into the mitochondria[23]. In addition, Cpt1 has been shown to have an anti-apoptotic function[24].

Silencing FASN causes a decrease in palmitic acid synthesis leading to the induction of apoptosis and formation of ROS[25]. In addition, the inhibition of FASN also induces the accumulation of NADPH, which in turn promotes the activation of ROS-generating enzymes such as NOX[26]. The excessive production of ROS results in DNA and protein damage, thereby affecting the oocytes and embryos [27]. The analysis of ROS-related genes further confirmed FASN increases ROS generation.

Apart from its action on metabolism, FASN is also involved in a number of additional processes. Although FASN is always considered as a key synthase in fatty acid synthesis, sometimes FASN play a role rather than fatty acid synthesis process. The AKT was a known target of the FASN, and AKT was involved in several biological signal pathways. In this study, we checked the function of FASN in embryonic development. We found that the FASN affect the porcine early embryonic development mainly through the AKT-p53 pathway. Many studies have demonstrated that FASN regulates AKT, which is a known target of FASN[12, 28]. The inhibition of the AKT pathway causes a decrease in blastocyst hatching[29]. Therefore, we hypothesize that FASN affects embryonic development *via* the AKT pathway. p53, which is associated with apoptosis, is one of the downstream products of the AKT pathway. FASN inhibition effectively initiates apoptosis by enabling p53. Many studies have also demonstrated that p53 induces growth arrest and apoptosis after DNA damage[9, 30]. TUNEL staining and analysis of apoptosis-related gene expression confirmed the effect of FASN on apoptosis. Furthermore, the co-localization of cytochrome C and the mitochondria showed that FASN inhibition induces the release of cytochrome C from the mitochondria. Many apoptotic factors stimulate the release of cytochrome C, which, in turn, initiates the apoptosis pathway[31]. Studies have provided insight into the crosstalk between apoptosis and autophagy. The anti-apoptotic proteins belonging to the BCL2 family bind Beclin-1 to suppress autophagy. Moreover, the caspase-mediated cleavage of autophagy-related proteins inhibits autophagy[32]. Our results demonstrate that FASN inhibition induces apoptosis, leading to the initiation of autophagy.

Many studies have described the relationship between FASN and proliferation. FASN-dependent FA synthesis is required for cell proliferation[26, 33]. FASN inhibition causes NADPH accumulation, which plays an important role in sustaining cell growth and proliferation[34]. Extracellular ATP-induced proliferation also requires the AKT pathway[35]. During embryonic development, FASN inhibition can also reduce cell proliferation. However, the mechanism by which FASN influences cell proliferation needs further study.

AKT is not only an anti-apoptosis and cell survival factor, but also a key regulator of pluripotency[18, 36]. AKT-mediated phosphorylation of *Oct4* is related to the proliferation of stem-like cancer cells[37]. As expected, FASN inhibition yielded a faint staining for OCT4 and a decrease in *Oct4* mRNA levels. In addition, apoptosis also generates a decrease in OCT4 expression[38].

miRNAs are non-coding RNAs that regulate many biological processes, including embryonic development [39–42]. miRNAs play an important role in the maternal-conceptus interface[43]. FASN inhibition causes changes in the expression of two miRNAs, miR-19 and miR-let7a. Both miR-92 and miR-let7a show decreased expression in blastocysts from male factor infertility or polycystic ovaries compared with blastocysts obtained from fertile women[44]. Additionally, let-7a can regulate the implantation potential of activated blastocysts[45].

**Fig 8:**
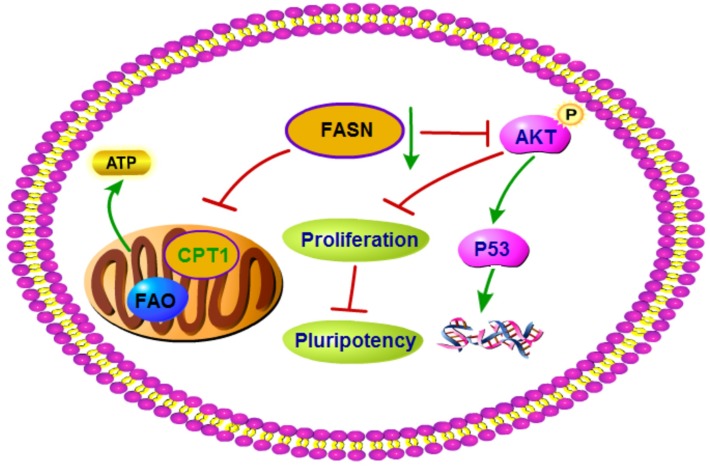
Schematic representation of the function of FASN during porcine early embryonic development. FASN is essential for the embryonic development in pigs. FASN impairment causes the inhibition of FAO through the regulation of Cpt1. Cpt1 is a key factor in the FAO process via its influence on the transport of FAs into the mitochondria. In addition, the inhibition of FASN can affect embryonic development through the AKT-p53 pathway, potentially inducing apoptosis. AKT can also influence cell proliferation through the regulation of pluripotency.

In conclusion, FASN plays a critical role in porcine early embryonic development. FASN exerts its embryogenesis function through two pathways. It provides ATP through FASN-dependent FAO and it affects apoptosis and survival through the AKT pathway (Fig 8).
